# Modeling and Simulation of Inter-Satellite Laser Communication for Space-Based Gravitational Wave Detection

**DOI:** 10.3390/s25041068

**Published:** 2025-02-11

**Authors:** Haoqian Liang, Zhaoxiang Yi, Hongling Ling, Kai Luo

**Affiliations:** 1 MOE Key Laboratory of TianQin Mission, TianQin Research Center for Gravitational Physics and School of Physics and Astronomy, Frontiers Science Center for TianQin, Gravitational Wave Research Center of CNSA, Sun Yat-sen University, Zhuhai 519082, China; lianghq29@mail2.sysu.edu.cn (H.L.); linghling@mail2.sysu.edu.cn (H.L.); 2 School of Electronics and Communication Engineering, Sun Yat-sen University, Shenzhen 518000, China

**Keywords:** space-based gravitational wave detection, inter-satellite laser communication, low-depth modulation, communication rate, bit error rate

## Abstract

Space-based gravitational wave detection uses an equilateral triangular satellite constellation with inter-satellite laser heterodyne interferometry to measure displacement variations caused by gravitational waves. Inter-satellite laser communication is critical for data transmission, redundancy and clock synchronization, which suppresses clock noise and enhances detection sensitivity. This integrated approach ensures precise gravitational wave information extraction, supporting the high-accuracy requirements of space-based observatories. This study focuses on the modeling and simulation of inter-satellite laser communication for space-based gravitational wave detection. Based on the data-transmission requirements of such systems, the principles of inter-satellite laser communication are analyzed. The research includes the selection of pseudo-random noise (PRN) codes, the signal scheme design and the development of the mathematical models for signal transmission. A simulation model is subsequently constructed in Simulink to evaluate the system. The simulation results confirm the accuracy of the model’s functionalities, including spreading, phase modulation, noise addition, phase demodulation and despreading. Additionally, the model achieves a data-transmission rate of 62.5 kbps with a bit error rate (BER) better than $10^{-6}$ when the modulation index exceeds $3.4\times 10^{-3}$, meeting the requirements for inter-satellite laser communication in space-based gravitational wave detection.

## 1. Introduction

In 1915, Albert Einstein proposed the theory of general relativity, which soon led to the theoretical prediction of gravitational waves [1]. To validate this prediction, physicists and astronomers worldwide began exploring methods to detect these ripples in spacetime. After decades of relentless effort, the LIGO achieved the first direct detection of gravitational waves in 2015 [2], capturing signals from a binary black hole merger event. This milestone marked the dawn of a new era in gravitational wave astronomy. Direct detection of gravitational waves relies on laser interferometers [3], which are broadly categorized into ground-based and space-based detection systems. In the realm of ground-based gravitational wave detection, the primary observatories currently include LIGO [2], Virgo [4], GEO600 [5] and KAGRA [6]. Ground-based detectors face limitations due to restricted arm lengths, ground vibrations and gravitational gradient noise, making them less effective at detecting low-frequency gravitational waves below 1 Hz [7]. However, the frequency range of 0.1 mHz to 1 Hz harbors a rich diversity of gravitational wave sources, promising valuable insights into astrophysical and cosmological phenomena [8]. To probe this low-frequency band, space-based gravitational wave detection has become a critical area of research.

Currently, the major space-based gravitational wave-detection missions include LISA [9], TianQin [10], Taiji [11], ASTROD-GW [12] and DECIGO [13]. From the perspective of the positioning of gravitational wave observatories, these space-based missions can generally be categorized into heliocentric, geocentric and libration configurations [14,15]. For heliocentric orbits, the relevant missions include LISA, Taiji and DECIGO. The LISA mission will deploy an equilateral triangular constellation with an arm length of 2.5 million km in a heliocentric orbit, approximately 20° ahead of Earth, as the gravitational wave observatory [16]. The Taiji mission will use a similar triangular constellation as the gravitational wave observatory, with a slightly larger arm length of 3 million km, positioned in a heliocentric orbit approximately 20° behind Earth [17]. The DECIGO mission will deploy four equilateral triangular constellations with the arm length of 1000 km in a heliocentric orbit as the gravitational wave observatory [18]. As for geocentric orbits, the TianQin mission is involved. The TianQin mission will place its gravitational wave observatory, a triangular constellation with an arm length of approximately 170,000 km, in a geocentric orbit at an altitude of about 100,000 km [19]. In terms of libration configuration, the ASTROD-GW mission is involved. In the ASTROD-GW mission, an equilateral triangle constellation with a side length of approximately 260 million km will be deployed at the Sun–Earth Lagrange points (L3, L4 and L5) as the gravitational wave observatory [20].

In space-based gravitational wave detection, the relative orbital motion of satellites, driven by orbital dynamics, causes continuous variations in the interferometer arm lengths, resulting in unequal arm configurations [21]. In laser interferometers, the frequency instability of the laser source constitutes a primary noise source, with its amplitude proportional to the arm length mismatch [22]. Taking LISA as an example, the interferometer arm length is $2.5\times 10^{9}$ m, while relative arm length variations due to orbital drift can reach up to $10^{5}$ km. To mitigate the laser frequency noise induced by these arm length variations, several stabilization techniques are employed. First, the laser frequency noise is suppressed to a pre-stabilized level of 30 $\mathrm{Hz}/Hz^{1/2}$ using the Pound–Drever–Hall (PDH) stabilization technique. Subsequently, it is further reduced to the $10^{-6}$$\mathrm{Hz}/Hz^{1/2}$ level through the combined application of the Arm-Locking Technique and Time-Delay Interferometry (TDI) [23]. In contrast, the TianQin mission requires the laser frequency noise to be suppressed to below 10 $\mathrm{Hz}/Hz^{1/2}$ after PDH stabilization and further reduced to the $10^{-5}$$\mathrm{Hz}/Hz^{1/2}$ level via TDI, satisfying the requirements for both measurement noise and relative arm length variations [24]. The fundamental principle of TDI is to reconstruct an equivalent interferometer with equal arm lengths by applying appropriate time delays and linear combinations to the measured phase data. This approach achieves common-mode suppression of laser frequency noise [25,26]. Therefore, the implementation of TDI requires precise measurement of the absolute distances between satellites to accurately reconstruct the equivalent interferometer. Additionally, the influence of clock noise in phase measurements necessitates data communication between satellites for clock noise elimination and onboard clock synchronization [27].

From a data-transmission perspective, space-based gravitational wave-detection missions are characterized by extended durations, with only one satellite in the constellation equipped to communicate directly with the ground. Consequently, the vast amount of data acquired through laser interferometry must first be transmitted bidirectionally across inter-satellite communication links and backed up by multiple satellites. At designated intervals, these consolidated data are transmitted to the ground by the designated satellite. This strategy minimizes the need for frequent antenna adjustments, thereby reducing disruptions to the gravitational wave-detection mission. It also provides a flexible and efficient solution for handling large-scale data transmission. Moreover, during the scientific measurement phase, a real-time inter-satellite communication link is essential within the constellation. This link enables the sharing of position and attitude data, facilitating the coordinated operation of all three satellites. Such synchronization ensures the collaborative control of satellite orbits and attitudes, which is critical for maintaining the precision and stability required for the successful execution of the gravitational wave-detection mission.

Inter-satellite laser communication for space-based gravitational wave detection presents unique challenges. The transmitting satellite employs Direct Sequence Spread Spectrum (DS/SS) technology to superimpose communication and ranging codes onto the laser link used for gravitational wave detection. This operation is constrained by the power distribution of the laser link, as only a small fraction of the total optical power can be allocated for communication and ranging to avoid interfering with gravitational wave detection. As a result, low-depth Binary Phase-Shift Keying (BPSK) modulation is adopted instead of the conventional BPSK modulation typically used in traditional inter-satellite laser communication. In space-based gravitational wave-detection missions, the optical power used for communication and ranging must be designed to not exceed 1% of the total optical power, meaning that the modulation depth should not exceed 0.1 rad [28].

Currently, in the research on inter-satellite communication and ranging for space-based gravitational wave detection, for the LISA mission, Esteban et al. have conducted studies on weak-light laser ranging and data communication using fiber optics and FPGA [28]. Under a modulation index of 0.1 rad, they achieved a data-transmission rate of 24.4 kbps with a BER of less than $10^{-3}$, along with an inter-satellite ranging resolution of 6 m. Considering the same modulation index, in hardware implementations by Baruse et al. [29], the data-transmission rate reached 78.125 kbps with a BER of less than $10^{-4}$, and the inter-satellite ranging resolution improved to 3.75 m. Similarly, with a modulation index of 0.1 rad, in experimental research by Yamato [23], a data-transmission rate of 78.125 kbps or 80 kbps was achieved, with a BER lower than $10^{-6}$ and an inter-satellite ranging resolution of 3.75 m. For the Taiji mission, in optical experimental studies by Peiquan Chen et al. [30]., using a modulation index of 0.1 rad, a data-transmission rate of 19.5 kbps with a BER of less than $10^{-6}$ and an inter-satellite ranging resolution of 3.75 m were achieved. Regarding the TianQin mission, Siyuan Xie conducted bidirectional laser ranging experiments [31], achieving an inter-satellite ranging resolution of 3 m.

From the existing published literature, in the area of inter-satellite laser communication and ranging for space-based gravitational wave detection, LISA has provided the requirement of communication data transmission and the mark of BER, proposed inter-satellite communication solutions based on ranging and conducted systematic experimental validation. Taiji has also proposed inter-satellite laser ranging and communication solutions and validated the optical systems. However, TianQin has only provided the requirement of data transmission and analyzed the performance of inter-satellite ranging. Whether TianQin’s inter-satellite communication performance can meet the requirements under different inter-satellite laser interferometric measurement parameters, compared to LISA, still needs to be analyzed. In the LISA constellation, the proposed inter-satellite communication solution requires a communication rate of no less than 15 kbps [29]. In the TianQin constellation, based on the optimized orbital design [32,33], the inter-satellite communication rate is required to exceed 41.54 kbps [34]. Meanwhile, for the LISA program, the target BER is less than $10^{-6}$ [23].

In response to these requirements, this paper will conduct an in-depth analysis of how to achieve high-reliability data transmission using lower power in gravitational wave-detection links, in order to meet the inter-satellite data transmission needs for space-based gravitational wave detection. The paper will focus on modeling and simulation of inter-satellite laser communication for space-based gravitational wave detection, and will also perform performance analysis of inter-satellite communication from a simulation perspective, based on the laser interferometry parameters of the TianQin constellation.

Section 2 of this paper introduces the basic principles and operational processes of inter-satellite laser communication for space-based gravitational wave detection. Section 3 outlines the simulation requirements, including the selection of PRN codes, the signal scheme design and the development of the mathematical models for signal transmission. Based on these requirements, a simulation model for inter-satellite laser communication is constructed using Simulink(R2023a). Section 4 validates the simulation model’s functionalities, including spreading, phase modulation, noise addition, phase demodulation and despreading. Additionally, it examines the system’s BER under various modulation indices, confirming that the simulation model meets the data-transmission requirements for inter-satellite laser communication in space-based gravitational wave detection.

## 2. Principles of Inter-Satellite Laser Communication for Space-Based Gravitational Wave Detection

**Figure 1:**
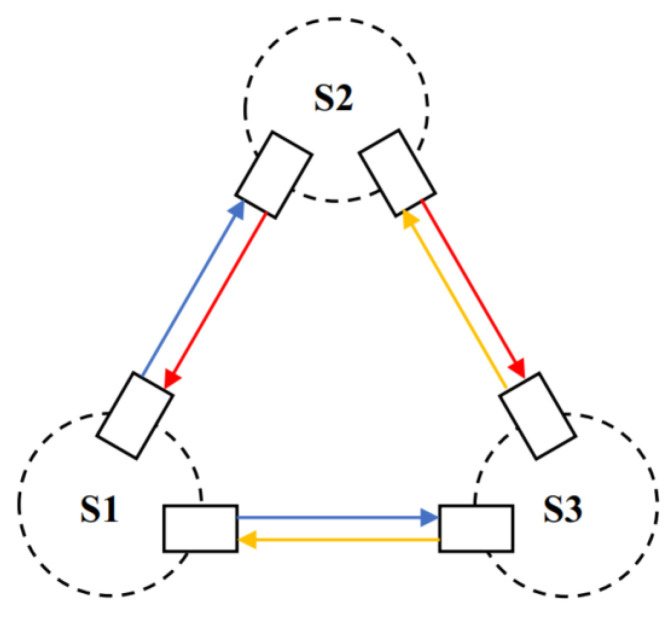
Schematic diagram of the three–satellite laser links for space–based gravitational wave detection.

In space-based gravitational wave-detection missions, the detection satellites form a three-satellite constellation configured in an equilateral triangular formation. Each arm of the triangle is equipped with bidirectional laser links, enabling the exchange of laser beams between satellites, as illustrated in Figure 1.

In the satellite constellation, individual satellites are represented by circular shapes with dashed lines. The laser emitters on the satellites are represented by rectangular boxes with solid lines. The laser emitted by satellite 1 (S1) is indicated by a blue arrow. The laser emitted by satellite 2 (S2) is indicated by a red arrow. The laser emitted by satellite 3 (S3) is indicated by a yellow arrow.

**Figure 2:**
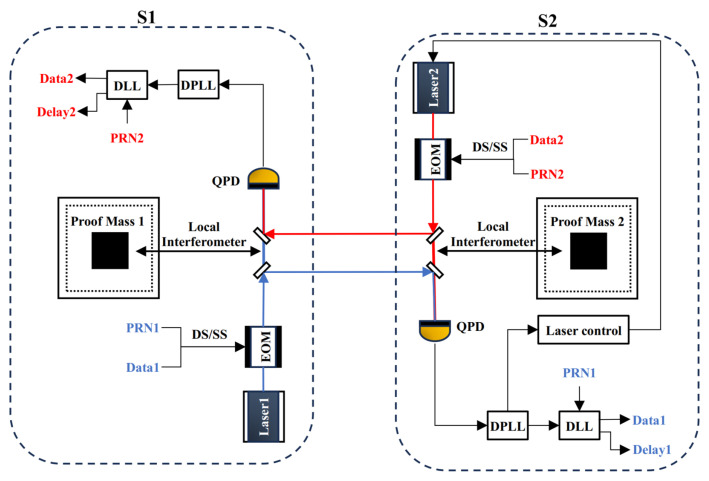
Schematic diagram of the principle of space–based gravitational wave detection.

In this equilateral triangular constellation, each satellite houses two square test masses that are completely isolated from external disturbances and suspended internally. Gravitational waves induce variations in the optical path length between two points in space. By employing a responder-based inter-satellite laser interferometry technique, interferometric measurements are conducted on the test masses located within different satellites. This process enables the detection of phase variations in the laser signals between the test masses, thereby allowing precise measurement of optical path changes. To illustrate, Figure 2 demonstrates the principle of gravitational wave detection using two satellites as an example.

Each satellite in the constellation is equipped with an independent frequency-stabilized laser. Taking S1 as the transmitting terminal, the laser emitted by the frequency-stabilized laser onboard S1 is expanded through a telescope and transmitted over hundreds of thousands of kilometers to S2. At the receiving terminal, the incoming weak laser is first heterodyned with the local laser emitted by the frequency-stabilized laser onboard S2 on its optical platform. The resulting interference signal is then detected by a quadrant photodetector (QPD), which converts the optical signal into an electrical signal. This electrical signal is processed using a digital phase-locked loop (DPLL) to extract the phase information of the interferometric signal. Subsequently, a weak-light phase-locking technique is employed to control the frequency-stabilized laser on S2, enabling it to emit a laser containing the phase variation information of the laser from S1. This laser is then transmitted back to the transmitting satellite, S1. At S1, the returning laser interferes with the local oscillator laser emitted by its onboard frequency-stabilized laser. The DPLL at S1 processes the resulting signal to extract the final phase information. This phase information contains the relative displacement variations of the test masses induced by gravitational waves, thereby enabling the detection of space-based gravitational waves.

In addition to its primary function of relative displacement measurement, the gravitational wave-detection constellation incorporates DS/SS techniques within its existing architecture. Using an electro-optic modulator (EOM), PRN code and data code are phase-modulated onto the laser via low-depth BPSK modulation. This approach facilitates absolute distance measurement and data transmission without compromising the picometer-level precision required for relative displacement measurements.

The DPLL and DLL (Delay locked loop), as key modules for implementing communication demodulation and data recovery, are designed and operate as follows:

(1) DPLL

**Figure 3:**
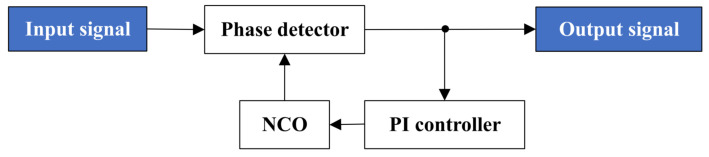
Structural schematic of the DPLL.

For the DPLL, it consists of a phase detector, a PI controller and a numerically controlled oscillator (NCO). The structural schematic is presented in Figure 3.

First, in the phase detector, the input beat note signal is compared in phase with the orthogonal signal output by the NCO, generating a phase error signal. The phase error signal is then passed through the PI controller to generate a control signal that adjusts the phase of the NCO. Next, the NCO generates the phase-adjusted orthogonal signal, which is once again phase-compared with the input beat note signal in the phase detector. This process repeats, and when the DPLL achieves loop locking, it enters a stable operating state. At this point, the signal output by the phase detector is the demodulated communication ranging code.

(2) DLL

**Figure 4:**
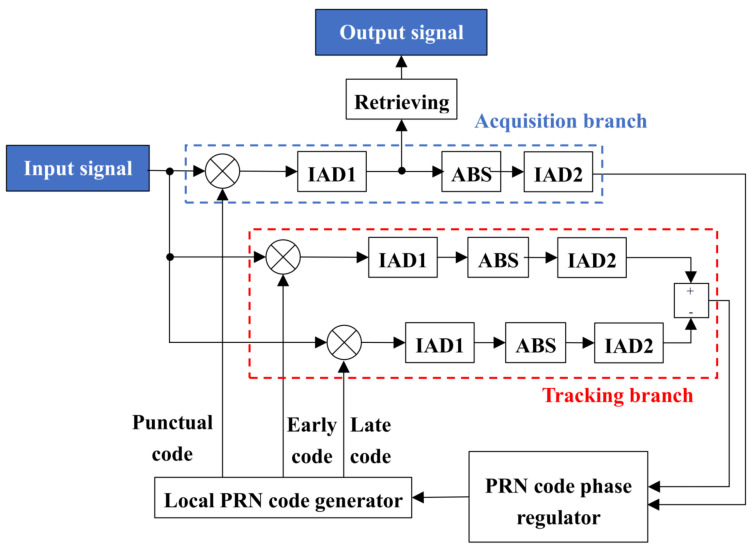
Structural schematic of the DLL.

For the DLL, it consists of an acquisition branch, a tracking branch, a PRN code phase regulator and a local PRN code generator. In the local PRN code generator, three PRN code signals are generated from the same PRN code sequence as the transmitter: the punctual code, the early code and the late code. The early code is shifted half a PRN chip period ahead of the punctual code, while the late code is shifted half a PRN chip period behind. The structural schematic of the DLL is shown in Figure 4.

**Figure 5:**
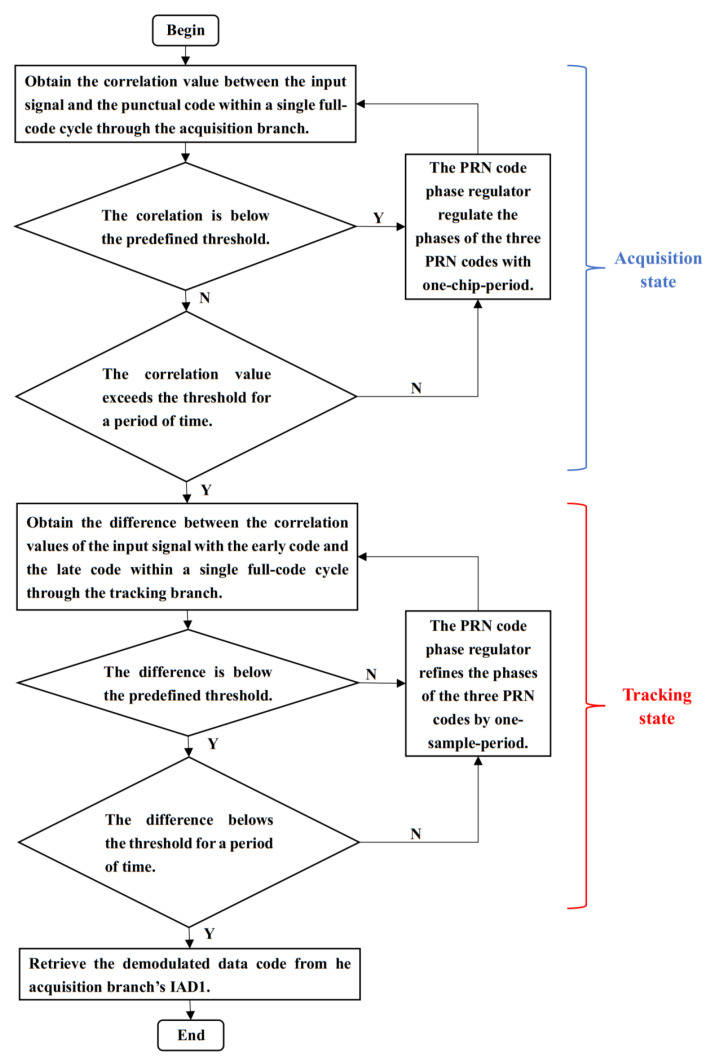
Flowchart of the DLL operation.

Upon receiving the demodulated communication ranging code signal from the DPLL, the DLL begins by performing acquisition. In the acquisition state, the input signal is first multiplied by the punctual code, followed by processing through the first-level integrator and dump filter (IAD1). In IAD1, integration is achieved by summing over multiple samples. Once the number of summations equals the number of data code samples, the integration resets. After IAD1 processing, the absolute value of the resulting signal is taken and passed to the second-level integrator and dump filter (IAD2) for further processing. Similar to IAD1, IAD2 performs integration by summing samples, but it resets after the number of summations reaches the total number of samples in one full code cycle. The output from IAD2 provides the correlation value between the punctual code and the input signal for each full code cycle, which serves as the control signal for the acquisition branch. When the correlation value from the acquisition branch is below the predefined threshold, the PRN code phase regulator will regulate the phases of the three PRN codes with one-chip-period precision. If the correlation value remains above the threshold for a sustained period, the acquisition is considered complete. At this point, the phase difference between the punctual code and the input signal is confined to within one PRN chip period. The acquisition process of the DLL can be seen in the acquisition state section of Figure 5.

Upon acquisition completion, the DLL transitions to the tracking state. In tracking, the input signal is multiplied by both the early and late codes. IAD1 and IAD2 process these multiplications to obtain the correlation values for each full code cycle. The difference between these correlation values controls the tracking branch. When this difference deviates from zero, the PRN code phase regulator refines the phases of the three PRN codes by one sample period. Once the difference remains zero or oscillates within a specific range, tracking is considered complete and the punctual code aligns with the input signal phase. The tracking process of the DLL can be seen in the tracking state section of Figure 5.

Once tracking is completed, the output signal from the acquisition branch’s IAD1 is processed to retrieve the demodulated data code after accumulating a number of times equal to the sampling points in a data code. Thereby, the the demodulated data code is obtained.

## 3. Inter-Satellite Laser Communication Model Based on Simulink

### 3.1. PRN Codes Selection

PRN codes are periodic sequences that can be deterministically reproduced and are widely used in communication and ranging systems. In conventional GPS satellite spread-spectrum communication systems, commonly used PRN code types include m-sequences and Gold sequences. These codes possess distinct characteristics, such as favorable autocorrelation and cross-correlation properties, making them suitable for diverse applications. However, in space-based gravitational wave-detection missions, the selection of PRN codes must address specific requirements and challenges.

In such missions, each satellite is equipped with two frequency-stabilized lasers, and laser beams are exchanged between the three satellites, forming a total of six laser links within the constellation. On each laser link, the DS/SS modulation scheme is employed. The data code and PRN code are XORed to generate a communication ranging code. This code is subsequently phase-modulated onto the laser link using low-depth BPSK modulation, facilitating simultaneous ranging and data communication. Given these operational requirements, six orthogonal PRN codes are necessary to ensure independent communication on each laser link without interference.

Selecting such codes not only facilitates robust tracking of individual PRN code amidst interference from adjacent laser links but also prevents cross-interference between different PRN codes. The autocorrelation properties of the selected PRN codes are crucial for precise ranging and reliable communication, while their cross-correlation properties play a significant role in minimizing interference. Therefore, the careful design and selection of six orthogonal PRN codes are critical for achieving the stringent communication and ranging requirements of space-based gravitational wave-detection missions.

**Figure 6:**
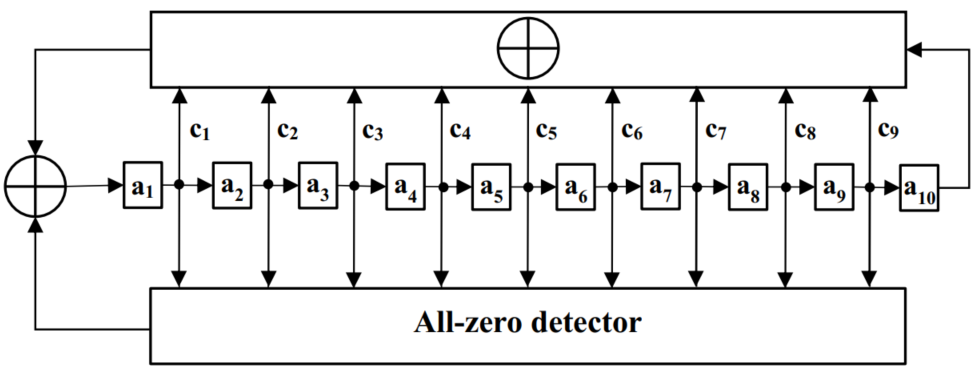
Schematic diagram of the M–sequence generator.

As for the selection of the PRN code type, we will consider the autocorrelation and cross-correlation properties of the PRN code, the flexibility of data code rate selection and the practical hardware implementation. In this regard, we have investigated m-sequences, Gold sequences and M-sequences. From the investigation of PRN codes, it is known that m-sequences and Gold sequences are generated based on linear feedback shift registers, while M-sequences are generated based on nonlinear feedback shift registers. When using an n-stage shift register to generate PRN codes, the resulting m-sequences, Gold sequences and M-sequences exhibit similar performance in terms of autocorrelation and cross-correlation. However, the lengths of the resulting PRN codes differ. The lengths of m-sequences and Gold sequences are $2^{n}-1$, while the length of M-sequences is $2^{n}$. For a length of $2^{n}-1$, there are fewer factors corresponding to the code length, which limits the available spreading factors in the spreading process and significantly restricts the flexibility of data code rate selection. In contrast, for a length of $2^{n}$, there are more factors, providing more available spreading factors in the spreading process and greatly enhancing the flexibility of data code rate selection. Additionally, because hardware implementation is based on digital circuits, and digital circuits are designed based on binary numbers, a code length of $2^{n}$ is also advantageous for practical hardware implementation. Considering these factors, for the selection of the PRN code in the space-based gravitational wave-detection task, this paper will choose an M-sequence with a length of $2^{n}$. The number of stages will be 10, and its structure is shown in Figure 6.

In the schematic, $a_{1}$ to $a_{9}$ represent shift registers, while $c_{1}$ to $c_{9}$ denote the feedback coefficients, indicating whether a given register participates in the feedback mechanism. Using this configuration, a 1024-chip even-length M-sequence is generated as the selected PRN code for the gravitational wave-detection mission. The 1024-chip length of the M-sequence offers significant advantages for data transmission. It allows the complete PRN cycle to be evenly divided into multiple data periods, thereby enabling higher data-transmission rates while maintaining the desired autocorrelation and cross-correlation properties necessary for reliable communication and ranging.

Although the structure of the M-sequence is predetermined, it exhibits statistical properties akin to random signals. For a random signal, its autocorrelation function is defined as:

(1) $$R_{a}\left(\tau \right)=\lim_{T\to \infty }\int_{-\frac{T}{2}}^{\frac{T}{2}}f(t)f(t-\tau )dt=\left\{\begin{array}{cc} 0 & \tau \neq 0\\ \mathrm{Constant} & \tau =0 \end{array}\right.$$

where τ is the time delay. T is the period of the random signal. f(t) is the time function of the random signal. According to Equation (1), when τ≠0, meaning f(t) and f(t−τ) do not overlap, $R_{a}\left(\tau \right)$ equals zero. Conversely, When τ=0, meaning f(t) and f(t−τ) completely overlap, $R_{a}\left(\tau \right)$ becomes a constant value.

The concept of cross-correlation, which quantifies the similarity between two different signals, is also relevant here. The cross-correlation function for two random signals can be expressed as:

(2) $$R_{ab}\left(\tau \right)=\lim_{T\to \infty }\int_{-\frac{T}{2}}^{\frac{T}{2}}f_{1}\left(t\right)f_{2}\left(t-\tau \right)dt$$

The M-sequence shares these properties, which enables the alignment of waveform and phase by performing autocorrelation between the received signal and a locally generated replica of the same PRN code.

**Figure 7:**
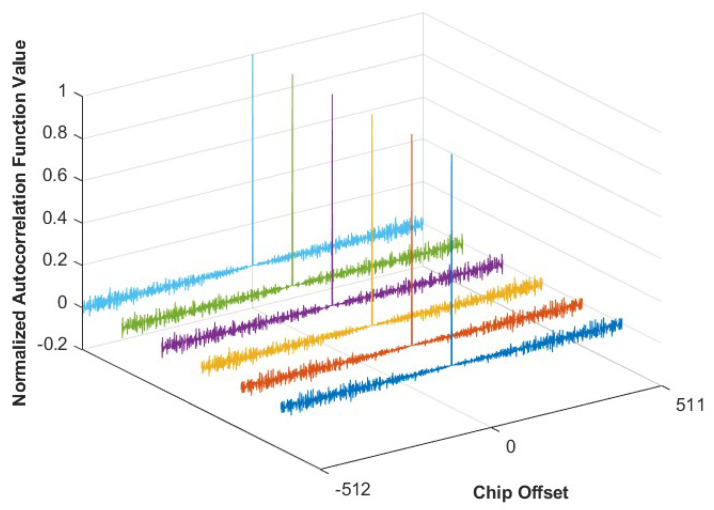
Normalized autocorrelation function values of M–sequences with different code offsets.

By employing different primitive polynomials, multiple distinct M-sequences can be generated. The normalized autocorrelation function values of the six selected M-sequences are shown in Figure 7. Since the length of the 10-stage M-sequence is 1024, the autocorrelation function values will be analyzed for chip shifts ranging from −512 to 511.

As shown in Figure 7, the autocorrelation values of the generated M-sequences exhibit a sharp peak only when perfectly aligned, i.e., when the chip offset is zero. At other delay times, the autocorrelation values are nearly zero, demonstrating the sequences’ excellent time-alignment properties.

**Figure 8:**
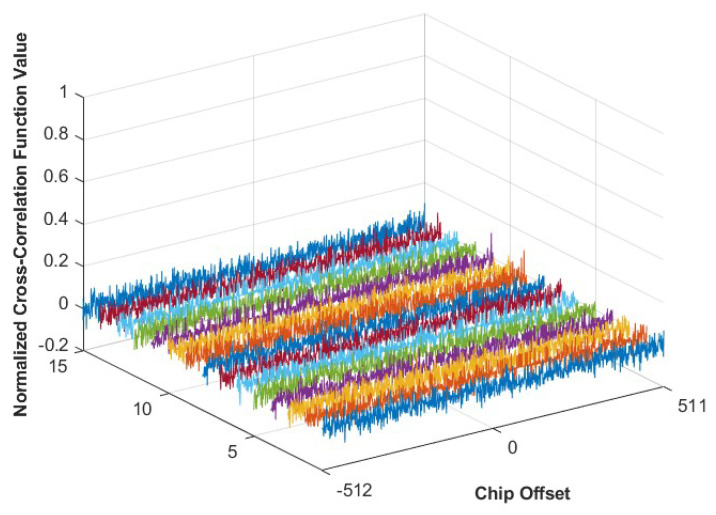
Normalized cross–correlation function values of M–sequences with different code offsets.

The normalized cross-correlation function values of the generated M-sequences are shown in Figure 8. Pairwise cross-correlation of the six M-sequences yields a total of 15 cross-correlation function values. Since the length of the 10-stage M-sequence is 1024, the cross-correlation function values will be analyzed for chip shifts ranging from −512 to 511.

As depicted in Figure 8, the cross-correlation values between any two of the six M-sequences are approximately zero for all chips offsets, confirming that the sequences are mutually uncorrelated.

In summary, the six selected orthogonal PRN codes demonstrate excellent autocorrelation and cross-correlation properties, fulfilling the stringent requirements for the gravitational wave-detection mission.

### 3.2. Signal Scheme Design

**Figure 9:**
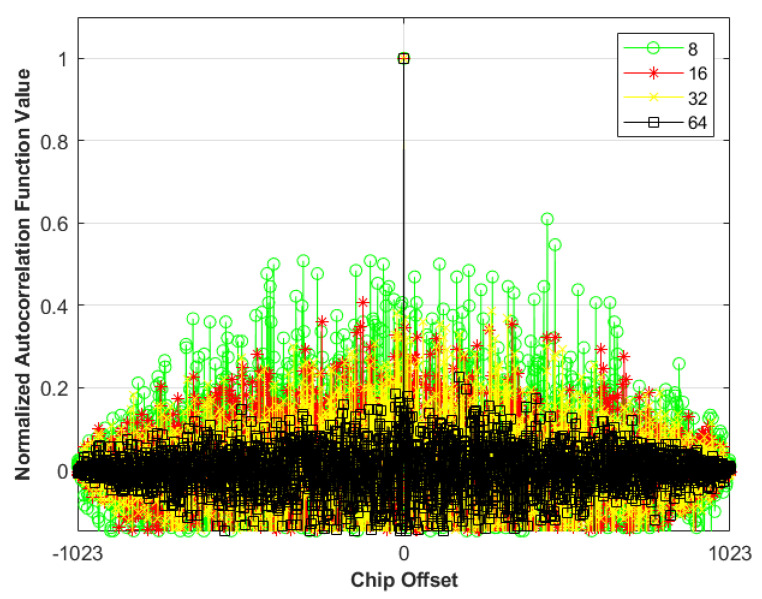
Normalized autocorrelation function values of PRN codes with spreading factors of 64, 32, 16 and 8.

To increase the data-transmission rate under a fixed PRN code rate, multiple data codes are incorporated into one complete PRN code cycle during the XOR operation. This process alters the autocorrelation properties of the resulting communication ranging code compared to the original PRN code. Therefore, it is essential to analyze how different spreading factors, corresponding to the number of data codes incorporated within a single PRN code cycle, affect the autocorrelation properties. The normalized autocorrelation function values of the PRN code at various spreading factors are shown in Figure 9.

**Figure 10:**
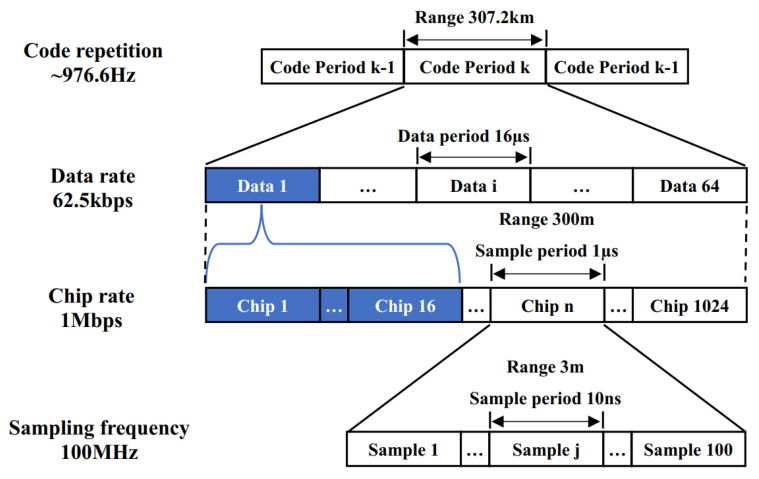
Design of the inter–satellite communication signal scheme.

As illustrated in Figure 9, reducing the spreading factor leads to poorer autocorrelation performance of the spread PRN code. To ensure that the signal scheme meets the inter-satellite communication rate requirements while maintaining satisfactory communication and ranging performance, a spreading factor of 16 is adopted for this study. Additionally, the higher the sampling rate, the better the inter-satellite ranging resolution. At the same time, a higher sampling rate also benefits signal retrieving, thereby aiding in the achievement of low bit error rates in communication. However, due to performance limitations in actual hardware, the maximum achievable sampling rate in current hardware development is 100 MHz. Therefore, a sampling rate of 100 MHz is selected for this case. The designed signal scheme is depicted in Figure 10.

In this scheme, the sampling rate is set to 100 MHz, with a PRN code rate of 1 Mbps and a data code rate of 62.5 kbps. The full code rate is approximately 976.6 Hz. Using a spreading factor of 16 and a PRN code length of 1024, each full code cycle corresponds to 1024 PRN codes and 64 data codes, with each data code encompassing 16 PRN codes.

### 3.3. Mathematical Model Analysis of Signal Transmission

Based on the principles of inter-satellite laser communication for space gravitational wave detection and the design of the inter-satellite communication signal scheme presented earlier, a mathematical model for the inter-satellite laser communication signal has been established.

At the transmitter, the PRN code signal is expressed as:

(3) $$u_{\mathrm{PRN}}\left(t\right)=\sum_{n=-\infty }^{\infty }a_{n}p\left(t-nT_{c}\right)$$

where $a_{n}$ represents the PRN code sequence, taking values of +1 or −1 with equal probabilities, and the pulse shape is given by p(t) with a period of $\frac{1}{T_{c}}$, where $T_{c}$ is the chip duration of the PRN code.

Similarly, the data code signal is expressed as:

(4) $$u_{\mathrm{data}}\left(t\right)=\sum_{i=-\infty }^{\infty }b_{i}p\left(t-iT_{d}\right)$$

where $b_{i}$ represents the data code sequence, with values of either 1 or 0, and the pulse shape is given by p(t) with a period of $\frac{1}{T_{d}}$, where $T_{d}$ is the symbol duration of the data code.

By performing DS/SS on the data code using the PRN code, the resulting spread signal is:

(5) $$u_{\mathrm{DS}}/\mathrm{SS}\left(t\right)=\sum_{i=-\infty }^{\infty }B_{i}\sum_{n=0}^{N-1}A_{n}p\left(t-nT_{c}-iNT_{c}\right)$$

where $B_{i}$ is the data code sequence after bipolar transformation, $A_{n}$ is the PRN code sequence after bipolar transformation (both taking values of +1 or −1) and *N* is the spreading factor, defined as $N=\frac{T_{d}}{T_{c}}$.

Letting $\sum_{n=-\infty }^{\infty }C_{n}=\sum_{i=-\infty }^{\infty }B_{i}\sum_{n=0}^{N-1}A_{n}$, the spread signal can be rewritten as:

(6) $$u_{\mathrm{DS}}/\mathrm{SS}\left(t\right)=\sum_{n=-\infty }^{\infty }C_{n}p\left(t-nT_{c}\right)$$

Here, $C_{n}$ is the communication ranging code sequence, and p(t) retains its periodicity with $\frac{1}{T_{c}}$.

The laser carrier for phase modulation is defined as:

(7) $$u_{\mathrm{carrier}}\left(t\right)=A_{c}\sin\left(\omega_{c}t\right)$$

where $A_{c}$ is the carrier amplitude, determined by the laser’s optical power. $\omega_{c}$ is the carrier angular frequency, determined by the laser’s optical frequency.

Combining Equations (6) and (7), the optical signal after low-depth phase modulation is given by:

(8) $$u_{\mathrm{BPSK}}\left(t\right)=A_{c}\sin\left(\omega_{c}t+m_{\mathrm{prn}}\sum_{n=-\infty }^{\infty }C_{n}p\left(t-nT_{c}\right)\right)$$

where $m_{\mathrm{prn}}$ is the modulation index applied for both communication and ranging.

At the receiver, let the time be $t^{\prime }=t-\tau$, where τ represents the signal delay due to transmission. Since the only terms of the Bessel function with a significant contribution in the optical amplitude are $J_{0}$ and $J_{1}$ [28]. After the laser used for communication, ranging and clock noise transmission undergoes heterodyne interference at the receiver, the resulting signal is given by:

(9) $$\begin{array}{cc} u_{\mathrm{HI}}\left(t^{\prime }\right)= & \;\eta G_{\mathrm{TIA}}J_{0}^{2}\left(m_{\mathrm{sb}}\right)\sqrt{\gamma P_{\mathrm{LO}}P_{S}}\sin\left(\omega_{\mathrm{het}}t^{\prime }+\varphi +m_{\mathrm{prn}}\sum_{n=-\infty }^{\infty }C_{n}p\left(t^{\prime }-nT_{c}\right)\right)\\ \; & +\eta G_{\mathrm{TIA}}J_{1}^{2}\left(m_{\mathrm{sb}}\right)\sqrt{\gamma P_{\mathrm{LO}}P_{S}}\sin\left(\omega_{\mathrm{sb}1}t^{\prime }+\varphi_{\mathrm{sb}1}\right)\\ \; & +\eta G_{\mathrm{TIA}}J_{1}^{2}\left(m_{\mathrm{sb}}\right)\sqrt{\gamma P_{\mathrm{LO}}P_{S}}\sin\left(\omega_{\mathrm{sb}2}t^{\prime }+\varphi_{\mathrm{sb}2}\right)+n\left(t^{\prime }\right) \end{array}$$

where η is the photodiode responsivity. $G_{\mathrm{TIA}}$ is the total gain of the transimpedance amplifier (TIA). $J_{0}\left(m_{\mathrm{sb}}\right)$ and $J_{1}\left(m_{\mathrm{sb}}\right)$ are the Bessel function of the first kind, which results from sinusoidal clock sideband modulation with an equivalent modulation index $m_{\mathrm{sb}}$. γ is the heterodyne efficiency. $P_{\mathrm{LO}}$ is the local oscillator optical power. $P_{S}$ is the signal optical power. $\omega_{\mathrm{het}}$ is the angular heterodyne frequency for the main carrier-to-carrier beat note. $\omega_{\mathrm{sb}1}$ is the angular heterodyne frequency for the lower sideband-to-sideband beat note. $\omega_{\mathrm{sb}2}$ is the angular heterodyne frequency for the upper sideband-to-sideband beat note. φ, $\varphi_{\mathrm{sb}1}$ and $\varphi_{\mathrm{sb}2}$ are the phases, which contain the science information. $n(t^{\prime })$ is a Gaussian white noise.

From a spectral perspective, the clock sidebands are located outside the main lobe of the main beat note frequency. Therefore, they do not affect the reception and modulation of the PRN code and can be neglected in the analysis of data transmission.

Neglecting the sidebands, the beat note signal after processing and ADC conversion becomes:

(10) $$\begin{array}{cc} u_{\mathrm{ADC}}\left(t^{\prime }\right)= & \;\sin\left(\omega_{\mathrm{het}}t^{\prime }+\varphi +m_{\mathrm{prn}}\sum_{n=-\infty }^{\infty }C_{n}p\left(t^{\prime }-nT_{c}\right)\right)+n^{\prime }\left(t^{\prime }\right) \end{array}$$

where $n^{\prime }\left(t^{\prime }\right)$ is also a Gaussian white noise.

### 3.4. System Simulation Model

**Figure 11:**
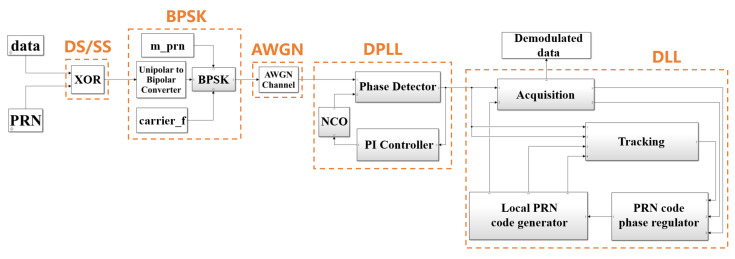
The simulation model of inter–satellite communication based on Simulink.

Building upon the previously analyzed principles of inter-satellite laser communication for space gravitational wave detection, a simulation model was developed using Simulink, as shown in Figure 11. The simulation model comprises the following key components: the DS/SS module, BPSK module, AWGN module, DPLL module and DLL module.

To streamline the simulation process, the laser phase modulation and heterodyne interference processes are simplified and represented as BPSK modulation. Within the model, the DS/SS, BPSK and AWGN modules are primarily responsible for generating the beat note signal, which serves as the input for the DPLL module.

## 4. Simulation Results and Analysis

### 4.1. Validation of the DS/SS Module

**Figure 12:**
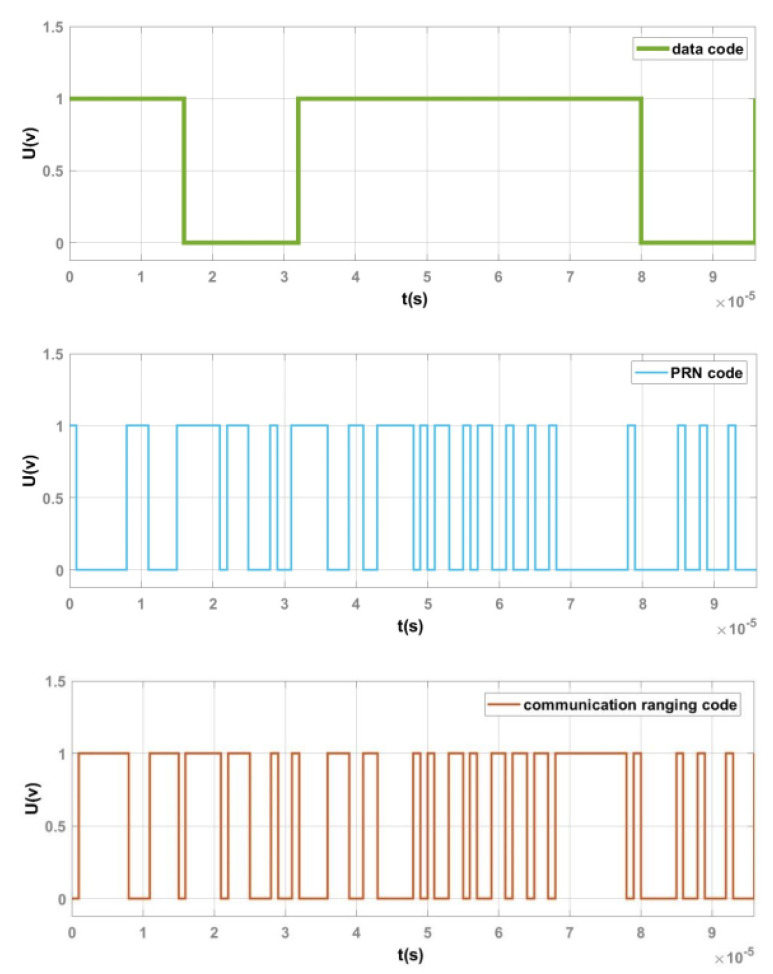
Time–domain waveforms of the data code, PRN code and communication ranging code.

**Figure 13:**
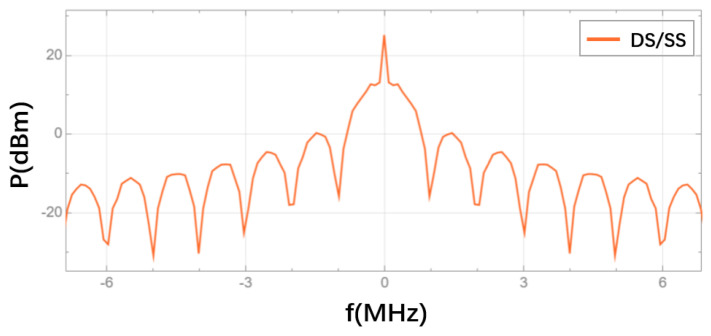
Spectrum of the communication ranging code.

In the DS/SS module, the time-domain waveforms of the data code, PRN code and the communication ranging code obtained after DS/SS are shown in Figure 12. The spectrum of the communication ranging code is presented in Figure 13. Since DS/SS effectively performs an XOR operation between the data code and the PRN code, the resulting communication ranging code will be as follows: when the data code is 1 and the PRN code is 0, the resulting communication ranging code will be 1; when the data code is 1 and the PRN code is 1, the resulting communication ranging code will be 0; when the data code is 0 and the PRN code is 1, the resulting communication ranging code will be 1; and when the data code is 0 and the PRN code is 0, the resulting communication ranging code will be 0. In other words, when the data code is 1, the communication ranging code should be the inverse of the PRN code; when the data code is 0, the communication ranging code should be identical to the PRN code. Meanwhile, spreading is essentially the process of expanding the frequency spectrum. Based on the inter-satellite communication signal system designed in Figure 10, it is clear that since the PRN code rate is 1 Mbps, after spreading, the main lobe bandwidth of the resulting communication ranging code in the frequency spectrum should be twice the PRN code rate.

As shown in Figure 12, when the data code is 1, the communication ranging code inverts the PRN code; when the data code is 0, the communication ranging code remains identical to the PRN code. This confirms that the data code and PRN code are subjected to XOR operation, consistent with the DS/SS principle. In Figure 13, the main lobe bandwidth of the communication ranging code spectrum is twice the PRN code rate, matching the theoretical spectral bandwidth after spreading. Therefore, the spreading functionality in the designed DS/SS module is correct.

### 4.2. Validation of the BPSK Module

**Figure 14:**
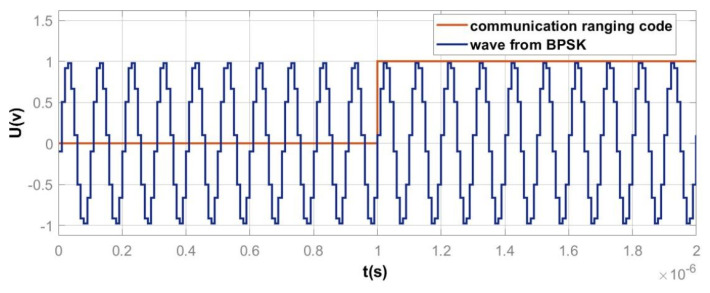
Comparison of the time–domain signals of the low–depth BPSK modulated signal and the communication ranging code.

**Figure 15:**
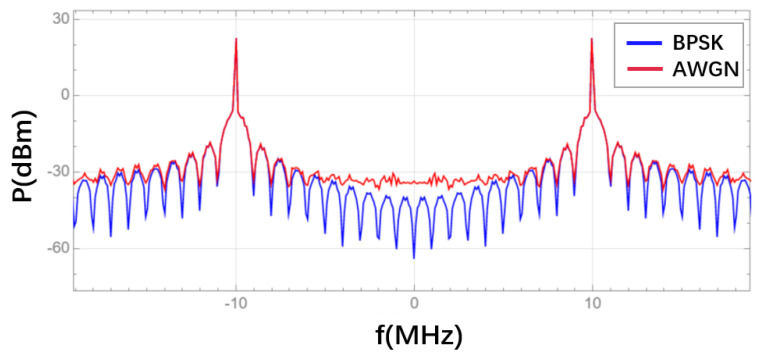
Comparison of the frequency spectrum of the signal after low–depth BPSK modulation and the frequency spectrum of the signal after noise addition.

In the BPSK module, the time-domain waveform and spectrum of the signal obtained after low-depth BPSK modulation are respectively shown in Figure 14 and Figure 15. In the BPSK module, the communication ranging code signal obtained from the DS/SS module needs to first undergo a conversion from unipolar to bipolar signals, i.e., transforming the sequence of 1 and 0 into a sequence of +1 and −1. Then, low-depth BPSK modulation is applied. By modulating the phase of the bipolar signal onto the carrier, the carrier can convey information by either leading or lagging in phase. Additionally, after phase modulation, a main lobe will appear at the carrier frequency, with a bandwidth equal to twice the PRN code rate.

As shown in Figure 14, when the communication ranging code is 1, the carrier phase shifts forward; when the communication ranging code is 0, the carrier phase shifts backward. Figure 15 shows that the spectrum of the signal after low-depth BPSK modulation exhibits sharp peaks at ±10 MHz, with the main lobes located in the ranges of −11 to −9 MHz and 9 to 11 MHz. This confirms that the communication ranging code, with a chip rate of 1 MHz, is successfully modulated onto a 10 MHz sine wave using low-depth BPSK modulation. Therefore, the phase modulation functionality in the designed BPSK module is correct.

### 4.3. Validation of the AWGN Module

In the AWGN module, the spectrum of the signal after the addition of Gaussian white noise is shown in Figure 15. Additive Gaussian white noise is uniformly distributed in the frequency spectrum. Therefore, adding additive Gaussian white noise to a signal will increase the power of the signal’s spectrum compared to its original state.

As shown in Figure 15, after noise addition, the power at the bottom of the signal’s spectrum increases slightly, which is caused by the added Gaussian white noise. Therefore, the noise addition functionality in the designed AWGN module is correct.

### 4.4. Validation of the DPLL Module

**Figure 16:**
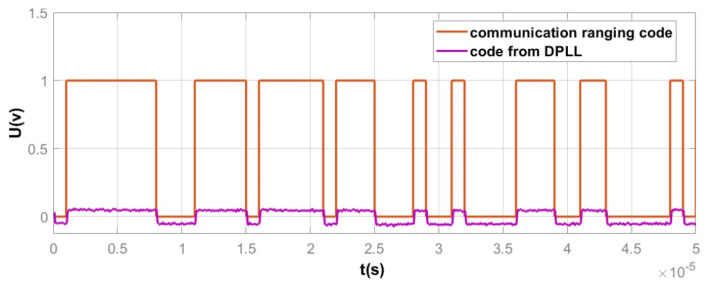
Comparison of the time–domain waveforms between the demodulated communication ranging code and the original communication ranging code.

In the DPLL module, the time-domain waveform of the communication ranging code obtained after demodulation is shown in Figure 16. In the DPLL module, due to the limitations of the designed DPLL performance, the demodulated communication ranging code will experience attenuation and will have residual noise that was not fully filtered out. Additionally, since the communication ranging code in the BPSK module is first converted into a bipolar signal before phase modulation, the demodulated communication ranging code in the DPLL module will also be a bipolar signal.

As shown in Figure 16, although the communication ranging code obtained after DPLL demodulation experiences attenuation and residual noise, its fluctuations remain consistent with the original communication ranging code. Therefore, the phase demodulation functionality in the designed DPLL module is correct.

### 4.5. Validation of the DLL Module

**Figure 17:**
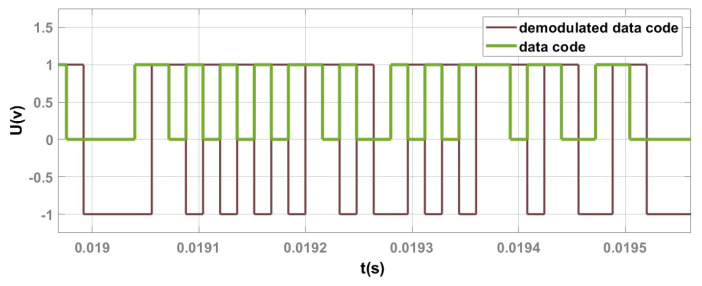
Comparison of the time–domain waveforms between the despread data code and the original data code.

In the DLL module, the time-domain waveform of the data code obtained after despreading is shown in Figure 17. In the DLL, once tracking is completed, the output signal from the acquisition branch’s IAD1 is processed to retrieve the demodulated data code after accumulating a number of times equal to the sampling points in a data code. As a result, the demodulated data code in the DLL module exhibits a one-data-period delay relative to the original data code, while maintaining consistency in fluctuations.

As shown in Figure 17, the data code after despreading matches the original data code, except for a one-data-period delay. This confirms the correctness of the spreading method and transmission process. Therefore, the despreading functionality in the designed DLL module is correct.

### 4.6. Bit Error Rate Analysis

**Figure 18:**
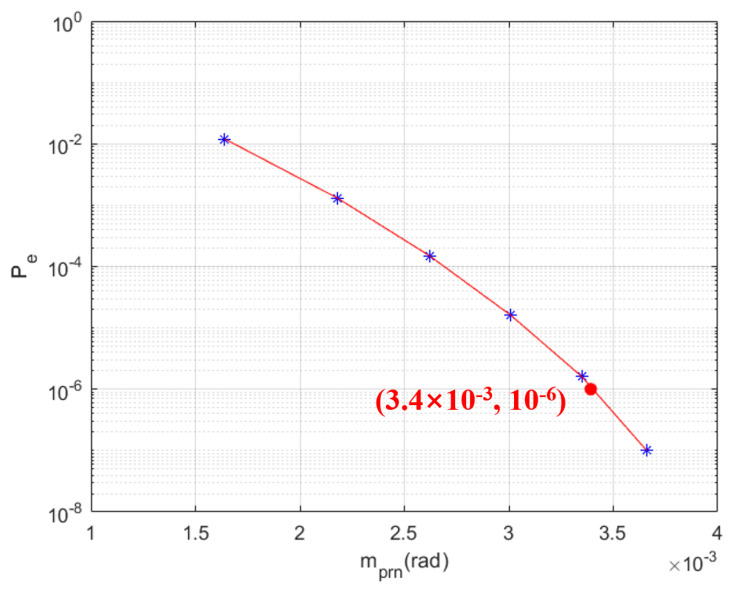
Bit error rate of the simulation system under different modulation indices and the corresponding fitted curve.

Based on the parameters of the TianQin constellation, the BER of the simulation system was analyzed for different modulation indices. The results and their corresponding fitted curve are shown in Figure 18.

From the fitted curve in Figure 18, it is evident that the BER is better than $10^{-6}$ when the modulation index exceeds $3.4\times 10^{-3}$. This indicates that the inter-satellite laser communication simulation system meets the requirements for space-based gravitational wave detection. Furthermore, all the measured BER data are based on the inter-satellite communication signal scheme designed in Figure 10, where the data rate for the transmission of the data code is 62.5 kbps. Therefore, the fitted curve corresponds to a data-transmission rate of 62.5 kbps.

## 5. Conclusions

This paper addresses the inter-satellite data-transmission requirements for space-based gravitational wave detection, analyzing key aspects of the inter-satellite laser communication system, including PRN code selection, signal scheme design and mathematical modeling of signal transmission. In PRN code selection, six orthogonal PRN codes with excellent autocorrelation and cross-correlation properties were chosen, satisfying the requirements of the gravitational wave-detection mission. For the signal scheme, the sampling rate was set to 100 MHz, the PRN code rate to 1 Mbps and the data code rate to 62.5 kbps, resulting in a total code rate of approximately 976.6 Hz. This design ensures the inter-satellite communication system meets the mission’s data rate requirements while providing good communication performance. A simulation model of inter-satellite laser communication was established using Simulink to validate the system design. The simulation results confirmed the proper implementation of spreading, phase modulation, noise addition, phase demodulation and despreading functionalities. Additionally, based on the TianQin constellation, the BER analysis demonstrated that the simulation model achieves a data-transmission rate of 62.5 kbps with a BER better than $10^{-6}$ when the modulation index exceeds $3.4\times 10^{-3}$, fulfilling the inter-satellite laser communication requirements for space gravitational wave detection. Future research will focus on refining the BER model, optimizing simulation results and improving data flow efficiency to enhance the performance of inter-satellite laser communication systems for space gravitational wave-detection missions.

## Data Availability

The original contributions presented in the study are included in the article; further inquiries can be directed to the corresponding author.

## Abbreviations

The following abbreviations are used in this manuscript:

PRN	Pseudo-random noise
BER	Bit error rate
PDH	Pound–Drever–Hall
TDI	Time-Delay Interferometry
DS/SS	Direct Sequence Spread Spectrum
BPSK	Binary Phase-Shift Keying
QPD	Quadrant photodetector
DPLL	Digital phase-locked loop
DLL	Delay locked loop
EOM	Electro-optic modulator

## References

[1] Einstein A., Rosen N. (1937). On gravitational waves. J. Frankl. Inst..

[2] Abbott B.P., Abbott R., Abbott T., Abernathy M., Acernese F., Ackley K., Adams C., Adams T., Addesso P., Adhikari R.X. (2016). Observation of gravitational waves from a binary black hole merger. Phys. Rev. Lett..

[3] Bond C., Brown D., Freise A., Strain K.A. (2016). Interferometer techniques for gravitational-wave detection. Living Rev. Relativ..

[4] Accadia T., Acernese F., Antonucci F., Astone P., Ballardin G., Barone F., Barsuglia M., Basti A., Bauer T.S., Bebronne M. (2011). Status of the Virgo project. Class. Quantum Gravity.

[5] Grote H., forthe LIGO Scientific Collaboration (2010). The GEO 600 status. Class. Quantum Gravity.

[6] Collaboration K. (2019). KAGRA: 2.5 generation interferometric gravitational wave detector. Nat. Astron..

[7] Hild S., Grote H., Degallaix J., Chelkowski S., Danzmann K., Freise A., Hewitson M., Hough J., Lück H., Prijatelj M. (2009). DC-readout of a signal-recycled gravitational wave detector. Class. Quantum Gravity.

[8] Gong Y., Luo J., Wang B. (2021). Concepts and status of Chinese space gravitational wave detection projects. Nat. Astron..

[9] Bayle J.B., Bonga B., Caprini C., Doneva D., Muratore M., Petiteau A., Rossi E., Shao L. (2022). Overview and progress on the Laser Interferometer Space Antenna mission. Nat. Astron..

[10] Luo J. (2022). The TianQin project. Proceedings of the The Fifteenth Marcel Grossmann Meeting: On Recent Developments in Theoretical and Experimental General Relativity, Astrophysics, and Relativistic Field Theories (In 3 Volumes).

[11] Ruan W.H., Guo Z.K., Cai R.G., Zhang Y.Z. (2020). Taiji program: Gravitational-wave sources. Int. J. Mod. Phys. A.

[12] Yang C., Zhang H. (2019). Formation flight design for a LISA-like gravitational wave observatory via Cascade optimization. Astrodynamics.

[13] Kawamura S., Ando M., Seto N., Sato S., Musha M., Kawano I., Yokoyama J., Tanaka T., Ioka K., Akutsu T. (2021). Current status of space gravitational wave antenna DECIGO and B-DECIGO. Prog. Theor. Exp. Phys..

[14] Xie X., Jiang F., Li J. (2024). Low Frequency Hierarchical Cooperative Impulse Control for Gravitational Wave Detector Formation Keeping. J. Guid. Control Dyn..

[15] Qiao D., Zhou X., Li X. (2023). Feasible domain analysis of heliocentric gravitational-wave detection configuration using semi-analytical uncertainty propagation. Adv. Space Res..

[16] Colpi M., Danzmann K., Hewitson M., Holley-Bockelmann K., Jetzer P., Nelemans G., Petiteau A., Shoemaker D., Sopuerta C., Stebbins R. (2024). LISA definition study report. arXiv.

[17] Luo Z., Wang Y., Wu Y., Hu W., Jin G. (2021). The Taiji program: A concise overview. Prog. Theor. Exp. Phys..

[18] Sato S., Kawamura S., Ando M., Nakamura T., Tsubono K., Araya A., Funaki I., Ioka K., Kanda N., Moriwaki S. (2017). The status of DECIGO. Proc. J. Phys. Conf. Ser..

[19] Mei J., Bai Y.Z., Bao J., Barausse E., Cai L., Canuto E., Cao B., Chen W.M., Chen Y., Ding Y.W. (2021). The TianQin project: Current progress on science and technology. Prog. Theor. Exp. Phys..

[20] Ni W.T. (2016). Gravitational wave detection in space. Int. J. Mod. Phys. D.

[21] Wang G., Ni W.T. (2019). Numerical simulation of time delay interferometry for TAIJI and new LISA. Res. Astron. Astrophys..

[22] Muratore M., Vetrugno D., Vitale S. (2020). Revisitation of time delay interferometry combinations that suppress laser noise in LISA. Class. Quantum Gravity.

[23] Yamamoto K. (2023). Intersatellite Clock Synchronization and Absolute Ranging for Gravitational Wave Detection in Space. Ph.D. Thesis.

[24] Ming M., Luo Y., Liang Y.R., Zhang J.Y., Duan H.Z., Yan H., Jiang Y.Z., Lu L.F., Xiao Q., Zhou Z. (2020). Ultraprecision intersatellite laser interferometry. Int. J. Extrem. Manuf..

[25] Wang G., Ni W.T. (2023). Revisiting time delay interferometry for unequal-arm LISA and TAIJI. Phys. Scr..

[26] Zhou M.Y., Hu X.C., Ye B., Hu S., Zhu D.D., Zhang X., Su W., Wang Y. (2021). Orbital effects on time delay interferometry for TianQin. Phys. Rev. D.

[27] Sweeney D. (2012). Laser Communications for LISA and the University of Florida LISA Interferometry Simulator.

[28] Delgado E., José J. (2012). Laser Ranging and Data Communication for the Laser Interferometer Space Antenna.

[29] Brause N.C. (2018). Auxiliary Function Development for the LISA Metrology System. Ph.D. Thesis.

[30] Chen P., Zhang Y., Deng R., Liu H., Luo Z. (2024). Experimental demonstration of bi-directional laser ranging and data communication for space gravitational wave detection. Results Phys..

[31] Xie S., Zeng H., Pan Y., He D., Jiang S., Li Y., Du Y., Yan H., Yeh H.c. (2023). Bi-directional PRN laser ranging and clock synchronization for TianQin mission. Opt. Commun..

[32] Ye B.B., Zhang X., Zhou M.Y., Wang Y., Yuan H.M., Gu D., Ding Y., Zhang J., Mei J., Luo J. (2019). Optimizing orbits for TianQin. Int. J. Mod. Phys. D.

[33] Ye B., Zhang X. (2024). Effects of lunisolar perturbations on TianQin constellation: An analytical model. Phys. Rev. D.

[34] Yi Z., Sun L., Jiang M. (2024). Data Transmission Analysis and Communication Scheme Design for TianQin Mission. IEEE Access.

